# Complete Genome Sequence of *Sulfitobacter* Phage ϕGT1, Isolated from a Tidal Flat

**DOI:** 10.1128/MRA.00779-20

**Published:** 2020-08-13

**Authors:** Chung Y. Hwang, Yirang Cho, Gwang I. Jang, Byung C. Cho, Stephen C. Hardies

**Affiliations:** a School of Earth and Environmental Sciences, Seoul National University, Seoul, South Korea; b Research Institute of Oceanography, Seoul National University, Seoul, South Korea; c Bioinformatics, Johns Hopkins University, Baltimore, Maryland, USA; d West Sea Fisheries Research Institute, National Institute of Fisheries Science, Incheon, South Korea; e Saemangeum Environmental Research Center, Kunsan National University, Kunsan, South Korea; f Department of Biochemistry and Structural Biology, University of Texas Health Science Center at San Antonio, San Antonio, Texas, USA; DOE Joint Genome Institute

## Abstract

The *Sulfitobacter* bacteria are ubiquitous and important players in organic sulfur cycling in marine environments. Here, we report the complete genome sequence of ϕGT1 infecting *Sulfitobacter* sp. HGT1, both of which were isolated from coastal sediment. ϕGT1 has a 40,019-bp genome containing 69 predicted protein-encoding genes.

## ANNOUNCEMENT

The genus *Sulfitobacter* belongs to the family *Rhodobacteraceae*, which is one of the major alphaproteobacterial groups in aquatic environments (1). *Sulfitobacter* bacteria have been isolated from diverse marine habitats and are known to affect the biogeochemical sulfur cycle in the ocean (1–5). Five *Sulfitobacter* phages have been isolated (Table 1), all of which are marine podoviruses (6–8).

**TABLE 1:** Genomic characteristics of ϕGT1 and other *Sulfitobacter* phages

Phage	Life cycle	GenBank accession no.	Genome size (bp)	GC content (%)	No. of ORFs	Coverage with ϕGT1 (%)	Identity with ϕGT1 (%)	Source or reference
ϕGT1	Lytic	MT584811	40,019	56.4	69	100	100	This study
NYA-2014a	Temperate	NC_027299	42,092	58.5	71	25	74.8	GenBank
ϕCB2047-A	Temperate	NC_020858	40,929	58.8	73	21	80.5	6
ϕCB2047-C	Temperate	NC_020856	40,931	59.0	71	21	78.2	6
ϕCB2047-B	Lytic	NC_020862	74,480	43.0	92	NS^*a*^	NS	7
EE36ϕ1	Lytic	NC_012696	73,325	47.0	79	NS	NS	8

a NS, no significant similarity.

The host bacterium *Sulfitobacter* sp. HGT1 and phage ϕGT1 were isolated from a tidal flat (37.62°N, 126.37°E) in the Yellow Sea, South Korea, in August 2011. A surface sediment sample was resuspended with a filtrate of ambient seawater that had been prefiltered with a 0.22-μm-pore membrane filter (Millipore). An aliquot of the slurry was spread on marine agar (Difco), and the plate was incubated aerobically at 30°C. Strain HGT1 was purified and identified as described previously (3). Phages in the filtrate (0.22-μm pore size) of ambient seawater were concentrated using Amicon Ultra-4 centrifugal filter units (Millipore). The phage concentrate was inoculated onto a host bacterial culture according to the double-layer plaque assay protocol (9). A single plaque, designated ϕGT1, was selected and purified by four rounds of double-layer plaque assays.

To extract genomic DNA, ϕGT1 concentrates were precipitated with polyethylene glycol 8000 (Sigma) from fully lysed plates as described previously (10). DNA was extracted using the QIAamp MinElute virus spin kit (Qiagen). A sequencing library was constructed with the Accel-NGS 2S PCR-free DNA library kit (insert size, ∼300 bp; Swift Biosciences) and sequenced by Macrogen, Inc. (Seoul, South Korea), using the Illumina MiSeq platform with a 2 × 300-bp paired-end format. Raw reads were processed with Trimmomatic version 0.39 (11) with default settings to trim the adapter and low-quality sequences. PhiX control reads were removed using the bbduk.sh script in the BBMap package version 38.84 (12). The trimmed reads (705,348 reads in total) were assembled using *de novo* assembly with default settings in CLC Genomics Workbench version 8.51 (Qiagen, Aarhus, Denmark), resulting in a circular contig with an average coverage of 3,795×. Phage termini and packaging mode were identified using PhageTerm with default settings (13). Open reading frames (ORFs) were predicted using Phage Search Tool Enhanced Release (PHASTER), GeneMark, GeneMarkS, and GeneMark.hmm (14–16). Translated ORFs were examined using PsiBLAST version 2.2.28+ and HHpred version 3.2.0 (17, 18) searches against the Protein Data Bank (PDB), Pfam, and NCBI conserved domain databases (19–21). Phylogenetic analysis of the large terminase sequence of ϕGT1 (phiGT1_2) was performed using the UCSC Sequence Alignment and Modeling system version 3.5 (22) and MrBayes version 3.2 (23), as described by Casjens et al. (24). The complete genome sequence of ϕGT1 was used as a query for MegaBLAST searching with default settings against the NCBI nonredundant/nucleotide database (release 237.0) to find highly similar sequences.

The host bacterium *Sulfitobacter* sp. HGT1 was most closely related to the type strain of Sulfitobacter marinus (25), based on the 16S rRNA gene sequence similarity (99.7%), with a GC content of 57.9%. The complete genome of ϕGT1 is 40,019 bp, with a GC content of 56.4% and a total of 69 predicted ORFs (Table 1). The ϕGT1 genome was concluded to be circularly permuted by a headful packaging system based on the results from the analysis of reads using PhageTerm and the similarity of the large terminase sequence to other headful packaging terminases. For reporting, the first base of the ϕGT1 genome was intentionally set at a start position of the gene encoding a putative small terminase subunit (phiGT1_1), the coding sequence of which typically contains the recognition and cleavage sites for the first end produced on the concatemer (26). The genome-wide comparison showed that the best hit for ϕGT1 was the temperate *Sulfitobacter* phage NYA-2014a in the *Podoviridae* family (Table 1). In terms of genome size and GC content, ϕGT1 was more similar to the three known temperate *Sulfitobacter* phages (40,929 to 42,092 bp, with GC contents of 58.5 to 59.0%) than the two known lytic *Sulfitobacter* phages (73,325 to 74,480 bp, with GC contents of 43.0 to 47.0%) (Table 1). Although ϕGT1 was distantly related to the three known temperate *Sulfitobacter* phages, with 21 to 25% coverage and 74.8 to 80.5% identity (Table 1), those three temperate phages were aligned among themselves over substantial fractions of their genomes (74 to 95%), with high identity values (99.5 to 99.9%). No significant match was found between ϕGT1 and the two known lytic *Sulfitobacter* phages (Table 1). ϕGT1 displayed a lytic life cycle based on plaque characterization (i.e., clear plaque formation) and gene content, including the presence of lysis genes (endolysin [phiGT1_23] and holin [phiGT1_24]) and the absence of an integrase gene. Neither terminal repeats nor RNA polymerase genes were found in ϕGT1, but ORFs homologous to podoviral core tail proteins P22 gp10 (phiGT1_15) and T7 tubular tail A (phiGT1_14) were found, indicating that ϕGT1 is likely to be a new marine lytic *Sulfitobacter* phage in the family *Podoviridae*.

### Data availability.

The annotated complete sequence of ϕGT1 has been deposited in DDBJ/ENA/GenBank under the accession number MT584811 (BioProject number PRJDB9847 and BioSample number SAMD00233255). The version of the phage genome described in this paper is the first version. The raw sequence reads are available in the DDBJ Sequence Read Archive with the accession number DRA010407. The genome sequence of the host bacterium *Sulfitobacter* sp. HGT1 is also available in DDBJ/ENA/GenBank under the accession number BLWI01000000 (BioSample number SAMD00228206).
